# Effect of timed exercise interventions on patient-reported outcome measures: A systematic review

**DOI:** 10.1371/journal.pone.0321526

**Published:** 2025-05-07

**Authors:** Mirey Karavetian, Cosette Fakih El Khoury, Femke Rutters, Romy Slebe, Diane Lorenzetti, Denis Blondin, André Carpentier, Jean-Pierre Després, Joris Hoeks, Andries Kalsbeek, Renée de Mutsert, Marie Pigeyre, Parminder Raina, Patrick Schrauwen, Mireille Serlie, Camilia Thieba, Jeroen van der Velde, David J.T. Campbell

**Affiliations:** 1 Faculty of Kinesiology and Physical Education, University of Toronto, Toronto, Canada; 2 National Institute of Public Health, Clinical Epidemiology, and Toxicology-Lebanon, Beirut, Lebanon; 3 Department of Epidemiology and Data Science, Amsterdam University Medical Center, Amsterdam, Netherlands; 4 Department of Community Health Sciences, Cumming School of Medicine, University of Calgary, Calgary, Canada; 5 Department of Medicine, Faculty of Medicine and Health Sciences, Université de Sherbrooke, Quebec, Canada; 6 Department of Kinesiology, Université Laval, Quebec City, Canada; 7 Department of Nutrition and Movement Sciences, Faculty of Health, NUTRIM School of Nutrition and Translational Research in Metabolism, Medicine and Life Sciences, University of Maastricht, Maastricht, Netherlands; 8 Department of Endocrinology and Metabolism, Amsterdam University Medical Centre, Amsterdam, Netherlands; 9 Department of Clinical Epidemiology, Leiden University and Medical Center, Leiden, Netherlands; 10 Department of Medicine, McMaster University, Hamilton, Canada; 11 Department of Medicine, Yale University, New Haven, Connecticut, United States of America; 12 Department of Interdisciplinary Health Sciences, Faculty of Health Sciences, University of Ottawa, Ottawa, Canada; 13 Department of Medicine, Cumming School of Medicine, University of Calgary, Calgary, Canada; 14 Department of Cardiac Sciences, Cumming School of Medicine, University of Calgary, Calgary, Canada; University Hospital Cologne: Uniklinik Koln, GERMANY

## Abstract

**Background:**

Exercising at a specific time of day has the potential to mitigate the negative effects of disrupted circadian rhythms caused by irregular work and sleep schedules on the development of chronic diseases. Afternoon/evening exercise is postulated to be superior to morning exercise for various health outcomes, but patient acceptance of timed exercise remains unclear. The aim of this systematic review was to assess the impact of exercise timing on patient-reported outcomes (PROMs).

**Methods:**

We conducted a systematic review, following Cochrane and PRISMA guidelines (PROSPERO: CRD42022322646). We systematically searched databases including MEDLINE, SCOPUS, Embase, APA PsycInfo, CINAHL, and Web of Science, to identify studies which reported on PROMs related to timed exercise interventions: either acutely after a bout of exercise or following extended training (>1 month). Studies were included if they reported primary data from randomized or non-randomized experiments of timed exercise interventions (against any comparator), published in English until August 2023 and reporting on any PROM. Machine-learning software (AR Reviews) was used to aid in abstract screening. Subsequently, two independent reviewers reviewed the included full texts, extracted study details (participants, interventions, outcomes), and evaluated the risk of bias using Cochrane tools (ROB-2 and ROBINS-I). Exercise interventions were summarized using the TIDieR reporting method and results were presented in accordance with the Synthesis Without Meta-analysis (SWiM) guidelines for systematic reviews.

**Results:**

Seventeen studies with 403 participants were included in the review. The interventions varied widely in exercise modality, duration, and participant characteristics, contributing to substantial heterogeneity in the findings. Most studies found no significant impact of exercise timing on PROMs. There was some inconsistency between studies for certain outcomes.

**Discussion:**

The review suggests that there are no clear detrimental effects of afternoon or evening exercise on PROMs compared to morning exercise. However, the lack of homogeneity in study populations and small sample sizes resulting in low power for PROM outcomes are major limitations of the research in this field. If future research confirms the metabolic advantages of afternoon/evening exercise, this may be an acceptable alternative for individuals.

## Introduction

Regular exercise is known to confer numerous health benefits and can be an effective tool in both preventing and treating many chronic medical conditions, including obesity and type 2 diabetes [1,2]. The global increase in chronic diseases is associated with the so called ‘Western Lifestyle’, which includes high calorie diets and sedentary behavior [3]. Moreover, disruptions in natural circadian rhythms caused by irregular work/sleep schedules, exposure to artificial light, and around-the-clock dietary intake are increasingly part of everyday life and are emerging independent risk factors for chronic diseases [4], and premature aging [2].

The specific timing of exercise has been shown to significantly modulate the circadian clocks and affect the body’s core clock and natural rhythms [3,5,6]. For example, exercising in the morning can advance the circadian rhythm, while evening exercise can delay it [3,7]. Thus, consistently scheduled exercise at certain times of the day may contribute to resetting the body’s circadian clock, and help to restore physiologic circadian rhythms [3], potentially aiding to prevent metabolic and other chronic diseases [3,8]. Afternoon exercise has gained some traction as being superior in improving metabolic derangements for those living with prediabetes or type 2 diabetes [1], or in those with excess body weight [9], and also in general populations [3,4,10]. Even though exercising at any time is widely believed to be better than not exercising at all, intentionally planning the timing of exercise may maximize the physiologic benefits derived from physical activity [4,8].

While consistently timed afternoon/evening exercise can be achieved in the setting of short-term clinical studies, it is unclear if this is sustainable over the long-term outside the experimental setting. Specifically timed exercise may impose burdens on individuals’ quality of life, making it essential to understand patient experiences and adherence outside research settings, as these aspects remain understudied and underreported.

It is well established that interventions which demand a great deal from patients, and lead to less satisfaction are unlikely to be taken up or maintained in daily life outside of research settings [11]. Health interventions and therapies that are clinically suitable and physiologically sound may not always be sustainable over the long-term, because patient perspectives of such interventions are not universally taken into account. Considering patients’ unique circumstances, beliefs, preferences and facilitators/barriers is important for increasing intervention acceptability and effectiveness.

Patient-reported outcome measures (PROMs) can be utilized to describe an individual’s personal, subjective evaluation of a given intervention based on its impact on their quality of life, symptoms, functioning, and physical, mental, and social wellbeing. PROMs can provide clinicians and researchers with unique information that cannot be obtained from purely biomedical measures. Understanding PROMs related to a certain treatment can help improve treatment protocols and facilitate mobilization of interventions into clinical practice and real-life situations and thus, achieve higher likelihood of adoption and adherence to the studied intervention [12]. Some studies incorporate pre- and post-intervention PROMs as pre-specified outcomes [13]. While there are many different PROMs, most are in the form of validated scales, some examples include measures of: health-related quality of life, depression, anxiety, pain, hunger, etc [13–16]. The aim of our systematic review was to identify and synthesize the findings from all PROMs that have been reported in interventional studies on timed exercise interventions.

## Methods

We conducted a systematic review and adhered to the Cochrane handbook guidance document [17] and the established reporting methods specified by the PRISMA (Preferred Reporting Items for Systematic Reviews) statement [18]. This review was part of a systematic review with a larger scope, with a protocol registered in the Prospective Register of Systematic Reviews (PROSPERO, registration number CRD42022322646; 3 May 2022. crd.york.ac.uk/prospero/display_record.php?ID=CRD42022322646). The broader review included the impact of a variety of different timed health behavior interventions (diet, exercise, sleep) on PROMs. Initially, the search aimed to identify the effect of all timed lifestyle interventions on PROMs. However, the large volume of relevant articles led to organizing studies into distinct categories of timed dietary interventions, timed exercise interventions, and sleep-related interventions. This approach allowed for separate systematic reviews of each intervention, enabling a more focused and detailed analysis of their effects on PROMs.

1. **Search methods for identification of studies**

- **Electronic searches**: We conducted a systematic literature search from inception until August 2023. Databases searched included: MEDLINE, SCOPUS, Embase, APA PsycInfo, CINAHL, and Web of Science. No date limits were applied. The search was conducted exclusively in English. Searches combined keywords and subject headings from three concepts: 1) patient perspectives (e.g., patient engagement OR patient perspective OR patient reported outcome measures OR patient reported experiences OR surveys OR qualitative research OR interviews OR focus groups); 2) diet, exercise or sleep (e.g., health promotion OR exercise/ physical activity OR diet/nutrition OR sleep OR behavior); and 3) timed interventions. The search strategy was formulated through comprehensive discussions between the primary authors and the health sciences librarian (DL), seeking to incorporate all variations of relevant keywords in the published literature into the search. The detailed search strategy can be found in the supplementary material (S1 Appendix).
- **Reference list searches**: The main literature search was supplemented by manually searching the reference lists from studies that met our inclusion criteria and systematic reviews on exercise.

1. **Study inclusion and exclusion criteria**

- **Types of studies**: We included all original interventional studies of any size that assessed timed exercise interventions, irrespective of comparator studied or randomization. Studies using retrospective and secondary data were excluded. Studies were excluded if they were dissertations, posters, conference abstracts, commentaries, reviews, letters to editors, or questionnaire development/validation programs.
- **Types of participants**: The review included studies in which human adults, or their surrogates reported a PROM related to timed exercise interventions. Studies conducted on children/adolescents or non-humans were excluded.
- **Types of interventions**: The review included all forms of timed exercise interventions, both acute exercise bouts and long-term training studies. Timed exercise interventions were defined as physical activity that was specified to be undertaken at a particular time of day. Studies were included if they included a comparison of any different timing of exercise [i.e., morning vs afternoon; morning vs evening; afternoon vs evening]. No restrictions were placed on the type, intensity and mode of exercise.
- **Types of outcome measures**: The review only included studies reporting one or more PROM – using either validated measures or bespoke tools.

1. **Screening and selection of literature**

- **Title and abstract screening**: Results of the search were imported into Endnote software for processing, including manual removal of duplicates and screening based on eligibility criteria. The cleaned list of references was imported into the ASReviews machine learning software [19] for title and abstract screening. ASReviews uses machine learning to rank the titles and abstracts on relevance, based on the patterns being established by the reviewer, and pre-specified articles of interest. A single reviewer (MK) conducted the initial screening using the software. The process for training and evaluating the AI tool was carefully conducted. Initially, we added 10 relevant and 10 irrelevant articles, clearly identifying them as such. After this initial training, the entire database of articles was input into the tool. From there, the reviewer (MK) continually marked each title and abstract as relevant or irrelevant. This interactive process allowed the tool to learn and refine its judgment, progressively surfacing the most relevant articles for review. This approach ensures that the tool becomes more accurate over time, although it does rely heavily on human input during the early training stages to establish a solid foundation for relevance assessment. The developers of the application recommend stopping the manual screening when 300 consecutive non-relevant articles have been reviewed and discarded. For precautionary reasons, we increased our margin to 500.
- **Full-text review and selection:** The full-texts from all articles identified as potentially relevant to the study at hand were reviewed in depth by two reviewers (MK and CF). Full-text articles were compared against predefined study eligibility criteria, to determine which studies were included and which were excluded. Agreement between the two reviewers was sought and disagreements were resolved through consensus or with the senior investigator (DJTC) as needed.

1. **Data management and analysis**

- **Data extraction:** Relevant data was extracted by two reviewers (MK and CF) in November and December 2023, using a pre-designed data extraction worksheet. Data included study details (author, year, design, duration), number and sex of participants, intervention description, and study outcomes related to PROMs. Details describing each exercise intervention followed the TIDieR method [20], to the extent permitted by the reported information.
- **Missing data:** We planned to do no imputation of missing data elements and planned to simply report where elements of methodology or reporting were lacking from each included study.
- **Assessment of risk of bias in included studies**: For Randomized Controlled Trials, we used Cochrane’s Risk of Bias 2 (RoB 2) tool to assess study quality [21]. The RoB 2 tool also has an extension for Crossover Trials [22], which we used for these specific study designs. Finally, for non-randomized studies of interventions, we used the ROBINS-I tool [23]. Risk-of-Bias assessments were conducted by two independent reviewers (MK and RS) with disagreements resolved through consensus discussions.
- **Unit of analysis issues**: Conversions were used to standardize units of measurement for homogeneity and comparability across studies.

1. **Data analysis and synthesis**

- **Data synthesis**: Results were reported following the guidelines of the Synthesis without meta-analysis (SWiM) in systematic reviews [24]. Assessing the certainty of the findings was not feasible given the varied nature of the PROMs reported by the different studies.
- **Measures of treatment effect**: Given the heterogeneity of PROM outcomes, a vote-counting method was applied [25]. Studies were classified based on whether they showed a reduction, no effect, or an increase in the outcome measure. Details of the treatment effect magnitude was also extracted.
- **Study grouping:** Because relevant PROMs may vary if the exercise is a single bout, or part of a longer-term program of physical activity, we felt that it was important to group the studies along these lines. We classified studies into acute exercise (assessing response to a single bout of exercise – which are most likely to impact immediate PROMs like exertion or fatigue) and long-term training (assessing incorporation of timed exercise over a sustained period of at least 1 month – most likely to impact PROMs like mental health, energy, etc…).

## Results

### Study selection and characteristics

**Fig 1:**
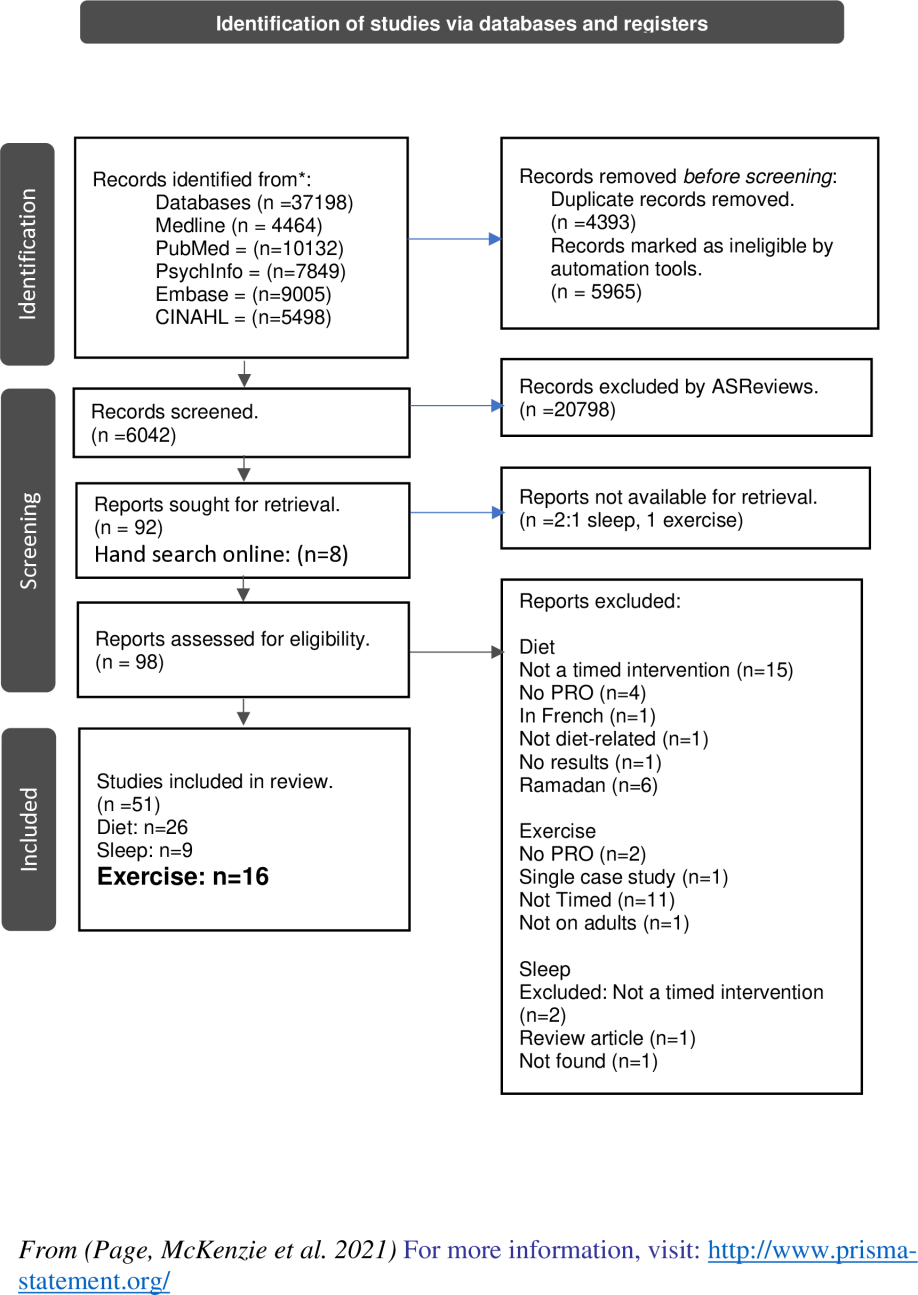
PRISMA flow diagram.

A total of 37,198 articles were located through database searches. Of these studies, 10,358 were removed when filtering for duplicates. ASReviews was used to screen 6042 titles/abstracts, after which we had reached 500 consecutive non-relevant articles and therefore stopped screening further studies using the software making the decision that all potentially relevant studies had been screened. Eight additional articles were identified through manual searches of reference lists. This resulted in 98 unique articles, of which 51 were included in the analysis after full text review. Of these, 26 were dietary interventions, 9 were sleep interventions and 16 on exercise interventions – only the latter were included in this review (Fig 1, S2 Appendix).

### Study design and risk of bias

Of the 16 timed exercise studies that are the focus of this review, we identified 10 cross-over RCTs, 5 parallel group RCTs and 1 non-randomized interventional study (Table 1). Details of included studies are reported in the supplementary material (S3 Appendix) and a summary of the main outcomes are shown in Table 2. Across all studies there was a total of 403 participants. The majority (n = 11) of manuscripts described acute exercise studies, with fewer studies (n = 5) examining the impact of sustained timed exercise (duration: 8–24 weeks) on PROMs. Most included studies were found to have moderate to serious risk of bias (Table 3). Details of the risk of bias assessment can be found in the supplemental material (S4 Appendix). One of the RCTs was evaluated to have serious risk of bias due to missing outcome data and 10 of the studies were noted to have some level of risk of bias where none of the studies reported a clear method of randomization nor the reasoning behind the reported results, which made them unlikely to be representative of the broader population.

**Table 1:** Study Characteristics and Sample description.

Reference	Sample	Design	Study Duration	Intervention	Outcome
**Acute Exercise Interventions**					
Azharuddin et al., [37]	Collegiate adults n = 20 Age: 23.20 ± 2.16 years BMI: 22.71 ± 3.07 kg/m2 Poor sleepers (Pittsburgh Sleep Quality Index >5) on; BMI < 30 kg/m2; I1: n = 10; I2: n = 10 Sex: M only	RCT (with apriori sample size)	1 day	I1: AM Exercise: 7h00 - 8h00 I2: PM Exercise: 19h00 - 20h00 Exercise protocol: 35–40 min exercise session • 5 min warm-up • knee extension (4 sets of 7 repetitions at 70% of 1RM) +10 min of cycling at 90% Vo2Max • 5 min cool-down.	Subjective Sleep Quality: using St Mary’s Hospital Sleep Questionnaire (SMHSQ) All PROMS are secondary outcomes of the study
Aloui et al., [31]	Healthy subjects n = 11 Age:21 ± 0.48 years BMI: 22.15 ± 0.54 kg/m2 Sex: M only	RCT Crossover	1 day per intervention, at least 36 hours washout period	I1: AM: 07:00 h I2: PM: 17:00 h Exercise protocol: Yo-Yo intermittent recovery test (YYIRT), 20min runs at a progressively increased speed. Each running bout is followed by a 10-s active recovery period	Rating of Perceived Exertion (RPE) Borg’s Rating (20-point scale) All PROMS are secondary outcomes of the study
McIver et al., [29]	Recreationally active men n = 12 Age: 25 ± 3 years Body fat: 21 ± 6% BMI: 26 ± 4 kg/m2 Sex: M only	RCT Crossover (with apriori sample size)	1 day per intervention, 7-day washout period in-between	4x5 h experimental trials: I1: AM Exercise: 8h00: AM-FASTED I2: AM Exercise: 8h00: AM-FED I3: PM Exercise: 15h00 PM-FASTED I4: PM Exercise: 15h00 PM-FED Exercise protocol: 45 min of brisk walking	Hunger, fullness, appetite, and food satisfaction: 100 mm Visual Analogue scale (VAS) Sleep Quality: PSQI: Pittsburgh sleep quality questionnaire All PROMS are secondary outcomes of the study
Mode et al., [28]	Healthy Participants. n = 16 Age: 25 ± 3 years BMI: 23 ± 2 kg/m2 Sex: F: 8, M: 8	RCT Crossover (with apriori sample size)	1 day per intervention, with at least 4 days washout period in-between	2 experimental trials I1: AM Exercise: 10:30 I2: PM Exercise: 18:30 Exercise protocol: a discontinuous incremental exercise on a cycle ergometer, involving 4-min incremental stages separated by approximately 5-min rest, performed until volitional exhaustion to determine VO2 peak	RPE: Borg’s Rating (20-point scale) Hunger, fullness, desire to eat (DTE), prospective food consumption (PFC), and nausea: VAS Primary outcome: Feelings of Appetite metrics
Bilski et al., [27]	Moderately active, non-smoking trainee firefighters n = 24 Age: 27.1 ± 3.1 years Height: 1.79 ± 0.1 m Weight: 76.1 ± 11.7 kg Sex: M only	RCT Crossover	1 day per intervention	I1: No Meal + Exercise at AM: 11:00 and PM: 23:00 on separate days I2: Meal + Exercise at AM: 11:00 and PM: 23:00 on separate days Exercise protocol: 30 second Wingate max exercise test	RPE: Borg’s Rating (20-point scale) Hunger ratings: 100mm VAS Appetite/Prospective consumption ratings: 100mm VAS Primary outcome: feeling of hunger
Benloucif et al., [33]	Older men and women n = 12 Age: 74.4 + - 5.5 years Healthy or with stable chronic medical conditions Sex: M: 4, F: 8	RCT Crossover	14 days per intervention; with 1-month wash-out period in-between	• I1:AM exercise: 9h00 - 10h00 • I2: PM exercise: 19h00 - 20h30 Exercise protocol: 30min physical activity (walking) + 30 min social interaction + 30 min mild to moderate physical activity (walk, dance..)	Subjective vigor and global mood: using 100 mm VAS Sleep quality: using Pittsburg Sleep Quality Index (PSQI) All PROMS are secondary outcomes of the study
Morita et al., [36]	Older participants with insomnia n = 40 I1: n = 12, age: 58.9 ± 3.6 y BMI: 22.2 ± 2.3 kg/m2 I2: n = 15, age: 57.7 ± 2.9 y BMI: 21.1 ± 1.8 kg/m2 Sex: I1: M: 5; F:10 I2: M:4; F: 8	RCT Crossover	1 day per intervention, with 14 days washout period in-between	50% of subjects were on AM morning exercise condition and the rest on PM exercise. I1:AM exercise: 09:30–11:00 I2:PM exercise: 17:30–19:00 Exercise Protocol: Step exercise (4 sets of 10 min)	Subjective sleep parameters: patient global impression of change scale (PGI-C) Primary outcome: Sleep
Li & Gleeson [32]	Healthy population n = 8 Age: 28.9 ± 1.8 y Sex: M only	RCT Crossover	1 day per intervention, with at least 4 days washout period in-between	I1: AM: 09:00 I2:PM: 14:00 h Exercise protocol: 2h cycling at 60% VO2max	RPE: Borg’s Rating (20-point scale). All PROMS are secondary outcomes of the study
Byers [41]	Participants from a rheumatoid arthritis outpatient clinic n = 30 Age: 61.9 ± 80.4 years Sex: M: 9, F:21	RCT Crossover	1 day per intervention, 2 consecutive days	All participants went though: Day 1: I1: AM + PM exercise, Day 2: I2: AM exercise only Exercise protocol: Non-weight-bearing, active range of motion exercises Time of evening and morning exercises were assigned as closely as possible to each patient’s usual bedtime and arising time.	Subjective Finger Stiffness: 1–10 (1 being severely stiff). All PROMS are secondary outcomes of the study
Kunorozva et al., [30]	Morning-type cyclists; n = 20 Age: 29.8 ± 7.7 years Sex: M only	RCT Crossover	2 weeks	Participants visited the lab 5 times at:06h00 10h00 14h00 18h00 22h00 (each on a separate day) Exercise protocol: Cycle for a total of 17 min on 3 different intensities categorized as stages: Stage 1: 6 mins - 60% HRmax Stage 2: 6 mins - 80% HRmax Stage 3: 3 mins - 90% HRmax 30 seconds pause between stages to recalibrate the cycle ergometer	RPE: Borg’s Rating (20-point scale) All PROMS are secondary outcomes of the study
Focht & Koltyn [34]	Recreationally trained men n = 21 Age: 21.4 ± 2.5 years Sex: M only	RCT Crossover (with apriori sample size)	3 day per intervention with a 72 hour washout period in-between	I1: AM RE: 6h00 & 8h00 I2: PM RE: 18h00 & 20h00 Exercise protocol: RE: 3 sets of 10 repetitions of 4 different exercises at 75% of individual’s 1 RM.	Pain ratings: 10-point pain rating scale at 3 times points: 1) pre RE, 2) RE + 1 min, 3) RE + 15 min State Anxiety: 20-item scale of the STAI (state-trait anxiety inventory). Primary outcome: Pain
**Long Term Exercise Interventions**					
Teo et al., [26]	Sedentary individuals; n = 40 Age: 51 ± 13 years BMI: 30.9 ± 4.2 kg/m2 I1: n = 20, I2: n = 20 Sex: M:17, F: 23	RCT (with apriori sample size)	12-week intervention	I1: AM Exercise: 8h00 – 10h00 I2: PM -Exercise: 17h00 – 19h00 I1 & I2 did 3 sessions/week Exercise protocol: Week 1–4: 30 min EE (70% Vo2 Max) + 30 min RE (45% 1RM) Week 5–8:30 min EE (70% Vo2 Max) + 30 min RE (50% 1RM) Week 9–12:30 min EE (70% Vo2 Max) + 30 min RE (55% 1RM) EE: threadmill walk	Appetite, Hunger & Disinhibition: 100 mm VAS Sleep quality: PSQI (Pittsburgh Sleep Quality Index) Daytime sleepiness: using Epworth Sleepiness Scale (ESS) All PROMS are secondary outcomes of the study
Küüsmaa-Schildt et al., [38]	Healthy, young participants n = 42 I1: n = 18, age: 33.5 ± 6.2 y, BMI: 25.7 ± 3.1 kg/m2 I2: n = 24, age: 31.4 ± 5.5 y, BMI: 24.3 ± 2.5 kg/m2 Sex: M only	Non-randomized control trial	24 weeks 1st training period: 12 weeks 2nd training period: 12 weeks	I1: AM exercise:0630–1000 h I2: PM exercise: 1630 h to 2000 h Exercise protocol: First 12 weeks: 2 combined training sessions of RE and EE. Second 12 weeks: 5 combined training sessions of RE and EE. Each exercise session: 60–120 min EE: 4 × 4 min high-intensity intervals (85–100%) separated by 4 min active resting periods (70% of HRmax). RE: leg press	Sleep Quality: using Pittsburg Sleep Quality Index (PSQI); higher scores indicate worse sleep quality. > 5 → poor sleep quality Fatigue: using 100 mm VAS Health-related quality of life (QoL); Bodily Pain and Vitality: using the Finnish RAND 36-item health survey All PROMS are secondary outcomes of the study
Seol et al., [40]	Healthy older adult participants n = 60, age: 71.0 + 3.9 y BMI: 23.7 + 4.0 kg/m2 I1: n = 30, age: 70.7 + 4.1 y BMI: 24.2 + 4.2 kg/m2 I2: n = 30, age: 71.3 + 3.8 y BMI: 23.1 + 3.8 kg/m2 Sex: I1: M: 8 F:22 I2:M:7 F: 23	RCT (with apriori sample size)	8 weeks intervention	I1: AM exercise: any time between waking up to 12:00 I2: PM exercise: any time between 18h00 to bedtime. Exercise protocol: Low-intensity EE for about 30 min every day at home	Subjective Sleep Parameters using sleep diary & PSQI score to measure Fatigue, Sleep efficiency, sleep satisfaction…) Primary outcome: Sleep
Saidi et al., [39]	Sedentary and inactive overweight/obese adult participants n = 28 I1: n = 16, age: 54.7 ± 8.5 y I2: n = 12, age: 53.5 ± 7.5 y Sex: I1: M: 4, F: 12 I2: M: 4, F: 8	RCT	12 weeks intervention	I1: Morning group (GM): 9:00 I2: Evening group (GE): 18:30 Exercise protocol: Moderate EE (60% HRmax) 3 × 90 min exercise session + RE (at 60% RM)/ week EE: Elliptical bike, rower, treadmill RE: targeted all major muscles	Sleep Quality: using Pittsburg Sleep Quality Index (PSQI) Habitual waking and bedtimes: using Morningness–eveningness questionnaire (MEQ) Daytime sleepiness: using Epworth Sleepiness Scale (ESS) Perceived fatigue and tiredness: using Pichot scale Health-Related Quality of Life (HRQoL): using Short Form Health Survey (SF-36) Primary outcome: Sleep
Passos et al., [35]	Sedentary adults - chronic primary insomnia diagnosis; n = 19 Age: 45 ± 1.9 years I1: n = 10, I2: n = 9 Sex: I1: M:2F:8 I2: M: 2, F: 7	RCT	6 months	I1: AM exercise: 10h00 ± 1 h – 3 days/week x 50 min I2: PM exercise:18h00 ± 1 h – 3 days/week x 50 min Exercise protocol: 3 days/week, 50 min treadmill 50 continuous at 60–70% of Vo2max	Quality of Life (QOL): using Short Form (SF-36) Questionnaire Scores Total Mood Disturbance (Tension-anxiety, Depression, Anger-hostility), using Profile of Mood States (POMS) Sleep quality: using Sleep Diary (7 day). Primary outcome: Sleep

M: Male; F: female; I: intervention; RPE: Rating of Perceived Exertion measured immediately post exercise, RE: resistance exercise, EE: Endurance Exercise, RM: Repetition Maximum, RCT: Randomized controlled trial

**Table 2:** Results of outcomes measures.

	Appetite			Pain and Fatigue				Sleep and Mood		Other
	Appetite	Hunger	Satiety & Fullness	Stiffness/ Pain	Rating of perceived exertion (RPE)	Energy/ Vigor/Fatigue	Quality of Life (QOL)	Sleep	Mood	0ther
**Acute Exercise Interventions**										
Azharuddin et al., [37]								↔		
Aloui et al., [31]					↑PM					
McIver et al., [29]	↓AM	↓AM	↔					↔		↔ (food satisfaction)
Mode et al., [28]		↔	↑AM		↔					↑ AM (nausea)
Bilski et al., [27]	↔	↔			↔					
Benloucif et al., [33]						↔		↔	↑AM (calmness)	
Morita et al., [36]								↔		
Li & Gleeson [32]					↔					
Byers [41]				↓PM						
Kunorozva et al., [30]					↑PM					
Focht & Koltyn [34]				↔					↔	
**Long Term Exercise Interventions**										
Teo et al., [26]	↔	↔	↔ (Disinhibition)					↔		
Küüsmaa-Schildt et al., [38]				↓AM		↔PM (Fatigue)	↑PM (Vitality)	↔		
Seol et al., [40]						↔		↑PM (satisfaction)		
Saidi et al., [39]						↔	↔	↔		
Passos, G. et al., [35]				↑PM (Bodily pain)			↑PM	↔	↔ (Depression, Anxiety)	↑ PM (Anger)

↔ : not significant (NS); ↓ : statistically significant (SS) decreases; ↑ statistically significant (SS) increases

**Table 3:** Risk of Bias for RCTs, cross over design and non-RCTs using the Cochrane tools.

Studies with non-randomized design	D1	D2	D3	D4	D5	D6	D7	Overall, Bias
Bias due to confounding	Bias in selection of participants into the study	Bias in classification of interventions	Bias due to deviations from intended interventions	Bias due to missing data	Bias in measurement of outcomes	Bias in selection of the reported results	
Küüsmaa-Schildt et al., [38]	–	**+**	**+**	**+**	**+**	**+**	**+**	–
**Studies with cross over design**	**D1**	**DS**	**D2**	**D3**	**D4**	**D5**	**Overall Bias**	
	Randomization process	Risk of bias arising from period and carryover effects	Deviations from intended interventions	Missing outcome data	Measurement of the outcome	Selection of the reported result		
McIver et al., [29]	?	**+**	**+**	**+**	**+**	?	?	
Focht, B. & Koltyn, K. [34]	?	**+**	?	**+**	**+**	?	?	
Kunorozva et al., [30]	?	**+**	**+**	**+**	**+**	?	?	
Bilski et al., [27]	?	**+**	?	**+**	**+**	?	?	
Benloucif et al., [33]	?	**+**	**+**	**+**	**+**	?	?	
Aloui et al., [31]	?	?	?	**+**	**+**	?	?	
Mode et al., [28]	?	**+**	?	**+**	**+**	?	?	
Li & Gleeson [32]	?	**+**	**+**	**+**	**+**	?	?	
Byers et al., [41]	?	?	**+**	**+**	**+**	?	?	
Morita et al., [36]	?	**+**	**+**	**+**	**+**	?	?	
**Studies with RCT design** [21]	**D1**	**D2**	**D3**	**D4**	**D5**	Overall Bias		
	Randomization process.	Deviations from intended interventions	Missing outcome data	Measurement of the outcome	Selection of the reported result			
Teo et al., [26]	**+**	**+**	**+**	**+**	?	**+**		
Seol et al., [40]	?	**+**	–	**+**	?	–		
Azharuddin et al., [37]	**+**	**+**	**+**	**+**	**+**	**+**		
Passos et al., [35]	?	?	**+**	**+**	?	?		
Saidi et al., [39]	?	?	?	**+**	?	?		

(-) indicated serious risk of bias, (?) indicated some concern, (+) indicated low risk of bias

### Impact on patient-reported outcomes (PROMs)

The most frequently studied PROMs that were evaluated in the included studies were: appetite (n = 4) (including hunger, satiety and fullness), perceived exertion (n = 6), mood (n = 4), sleep quality (n = 9), and joint pain/stiffness (n = 4). Other less frequently reported PROMs were vigor/fatigue, quality of life, and a variety of others including anger, calmness, and intrinsic motivation (Table 3).

#### Appetite.

One acute and one long-term exercise intervention study [26,27] reported that timing of exercise did not significantly affect hunger and appetite. Another acute exercise study examined the effect of exercise timing on participant-reported hunger and prospective food consumption, finding that with AM exercise, participants felt more satiated, with decreased desire to eat after exercise [28]. Another acute exercise study reported that fullness and food satisfaction were not significantly different between morning and evening exercise, while hunger and appetite decreased significantly with morning exercise compared to evening exercise [29].

#### Exertion.

We found inconsistent results concerning the impact of exercise timing on reported perceived exertion (RPE) during or after exercise sessions. One acute exercise study showed significantly higher levels of RPE during evening exercise among male cyclists who typically exercise in the morning [30]. A similar response to afternoon exercise was seen in a non-athletic population inclusive of both sexes [31]. However, RPE immediately after exercise was not influenced by exercise timing in acute exercise studies among moderately active male firefighters or healthy young adults [28,32].

#### Mood.

Three studies examined the impact of timed exercise on self-reported mood. An acute exercise study showed that morning exercise was associated with significantly higher feelings of calmness among elderly participants [33], but this effect was not reported in young recreationally trained males [34]. In a long-term exercise training study among middle aged adults, overall mood was unaffected by timing of exercise, except for feelings of anger that were found to be higher with PM exercise [35].

#### Sleep outcomes.

With regards to sleep, the majority of studies that examined this outcome reported no significant differences by exercise timing [26,29,33,35–39]. The only study that reported significant changes in sleep parameters was conducted by Seol et al. [40], whereby elderly individuals reported reduced sleep satisfaction following evening exercise.

#### Quality of life (QoL).

The impact of timed exercise interventions on QoL revealed mixed results and were reported in the long-term training exercise studies. In one study sedentary individuals with chronic insomnia had significantly better QoL scores with evening exercise [35], while sedentary and inactive overweight/obese adults showed no impact of exercise timing on QoL [39]. Vitality, a construct of QoL, was found to be significantly decreased in those who exercised in the morning as compared to the evening [38].

#### Pain.

Bodily pain and stiffness after exercise was evaluated in 2 acute exercise studies. Evening exercise decreased feelings of stiffness/joint pain among adults living with rheumatoid arthritis [41], yet another study showed that timing of activity had no impact on this variable for recreationally trained men [34]. However, in the long-term training studies, Passos et al. [35] showed higher reported bodily pain with evening exercise. Moreover, in a long-term exercise training study, healthy, young males, showed a lower reported bodily pain and fatigue with morning exercise, compared to evening exercise [38], when assessed by the health related QOL questionnaire.

## Discussion

The aim of this systematic review was to synthesize the evidence on the effect of exercise timing on PROMs. There is a relative paucity of research on this topic, as only 16 studies with a total of 403 participants were included in the review. Studies primarily investigated the effect of exercise timing on sleep quality (n = 8), with other PROMs explored less frequently. More than half of the reported PROMS were not significantly affected by the timing of exercise. While it is difficult to make any conclusive statements about the effects of these interventions due to the heterogeneity in the interventions and outcomes, in general, morning exercise seemed to have more positive associations with PROMs [28,29,33]. However, in two instances, certain PROMs were positively affected by evening exercise such as decreased stiffness [41], and improved vitality [38].

Studies included in our review found that exercise timing did not impact appetite [26] and hunger [27]. These findings are consistent with a review on the impact of exercise on appetite regulating hormones, which revealed that exercise suppresses appetite through stimulating anorexic hormones and this effect is not influenced by exercise timing [42].

Included studies were also inconclusive [27,28,32] for a clear effect of timing of exercise on perceived exertion, with 2 [30,31] out of 5 studies reporting significant increases in perceived exertion in evening exercisers, particularly notable in a study involving “early riser” cyclists whose evening exercise coincided with their habitual sleep time [30].

Literature has revealed that exercise is beneficial for mood and mental health outcomes [43]. Despite inconsistent effects of exercise timing on mood [33–35], one study found that morning exercise was associated with positive emotions when compared to afternoon or evening exercise [33]. Furthermore, the overall QoL score did not change due to exercise timing [38,39], but less bodily pain was reported with morning exercise in acute [35] and long-term [41] studies. Interestingly, evening exercise was found to reduce joint stiffness in adults with rheumatoid arthritis [41], potentially explained by the physiological changes controlled by the circadian rhythm, such as inflammatory cytokines and body heat that gradually improve mobility throughout the day [44]. Finally, the relationship between exercise timing and sleep parameters was mixed in our review. There is a robust body of evidence establishing a positive relationship between exercise and sleep in general [45] which often discourages exercising close to bedtime [46], for better sleep hygiene. However, in the included studies, exercise timing did not have significant impact on subjective sleep outcomes [26,29,33,35–37,39], apart from one study in older adults, who experienced better sleep satisfaction with evening exercise [40]. These findings align with a recent systematic review which indicated that there is no detrimental effect of evening exercise on sleep [10].

Overall, the studies included in this review were evaluated to have significant risk of bias, based on the Cochrane assessment tools. One common limitation for several of the non-randomized studies was the fact that the samples were small and conveniently chosen. Measures were often not taken to ensure population representativeness [36,38]. Furthermore, the PROMs reported in this review were largely secondary outcomes in studies that were powered to detect differences in biological parameters. Therefore, it is likely that the results of this review are underpowered to comment conclusively on the impact of exercise timing on PROMs. For enhanced understanding of the impact of timed behaviors on PROMs, future research should consider using PROMs as primary or co-primary outcomes and studies should be powered accordingly.

The articles included in this review analyzed the effects of exercise within controlled research settings, these designs are unable to fully consider the impact of timed exercise protocols in real-life situations, inclusive of social duties and work commitments. A drawback of the current body of research is that while consistent afternoon/evening exercise can be achieved in short-term clinical studies without significant detrimental effect on PROMs, it remains unclear if this would be viable for most people in the long run in the context of their everyday lives outside controlled experimental conditions. Moreover, the significant heterogeneity in exercise protocols, participant demographics, exercise duration, timing, and intervention lengths across studies, makes drawing firm conclusions challenging. The lack of standardization in these parameters highlights the need for more consistent research methodologies for reporting on PROMs in this field. Furthermore, the PROMs that have been collected to date do not explicitly explore participants’ acceptability of the intervention. To gain a comprehensive understanding of the practicality and long-term effectiveness of exercise interventions, future research should explore how timed exercise can be sustained in everyday life, taking into account individuals’ social duties and professuional commitments in addition to the kinds of PROMs that have been assessed to date. By addressing the challenges of real-life scenarios and adopting more standardized research methodologies, we may be able to draw more reliable conclusions about the sustainability of exercise timing interventions on long-term health and well-being.

The participants in the various studies covered a wide range of ages, and people with varying physical fitness backgrounds, and the included studies used different types of exercise interventions. This is a strength as it shows relative consistency in the findings, regardless of differences in these factors. Conversely, this may also be viewed as a limitation given that it makes direct comparison between the studies difficult. One limitation to the studies included is that approximately half of the studies included only male participants. Therefore, it is possible that the outcomes reported are not applicable to females and women, and future research is needed to understand potential sex and/or gender differences. More high quality RCTs (with lower risk of bias) investigating the effect of exercise timing on PROMs are needed. Other limitations of this review include the fact that only studies published in the English language were included, and that we were unable to complete a meta-analysis of the included studies given the marked heterogeneity in study design, intervention type, and outcome measures.

## Conclusion

In conclusion, this systematic review suggests that timed exercise interventions do not seem to have a consistent effect one way or the other on PROMs that have been assessed to date. Some studies suggested that evening exercise may have a positive effect on perception of sleep quality, when compared to morning exercise. On the other hand, other studies suggest that morning exercise may be beneficial in several domains such as reducing hunger and improving anxiety. Overall, the findings of this review showed mixed results on multiple patient-reported outcomes and thus results remain inconclusive. That said, however, there does not seem to be a clear detrimental effect of afternoon/evening exercise compared to morning exercise with respect to most PROMs – which means that if ongoing and future clinical studies prove its metabolic superiority, evening exercise may be acceptable to individuals without clear detrimental effect on PROMs.

## Supporting information

S1 Appendix Search strategy. (DOCX)

S2 Appendix Studies screened at title/abstract stage. (XLSX)

S3 Appendix List of studies reviewed at full-text stage and reasons for exclusion/inclusion. (DOCX)

S4 Appendix Detailed study characteristics and results. (XLSX)

S5 Appendix Risk of bias using Cochrane tools for randomized and non-randomized trials. (XLSM)
