# Supplementary material for: Effect of timed exercise interventions on patient-reported outcome measures: A systematic review
Source: PLoS One. 2025 May 7;20(5):e0321526. doi: 10.1371/journal.pone.0321526 (PMC12057914; doi:10.1371/journal.pone.0321526)
Supplement: S1 Appendix — (DOCX) [file pone.0321526.s001.docx]

### Appendix A: Timed health behavior change interventions: a systematic review of patient reported outcomes (PRO)

**Patient Reported Outcomes AND Health Behavior Intervention AND Timed Interventions**

**MEDLINE**

**Patient Reported Outcomes**

| exp Patient Satisfaction/ OR exp patient reported outcome measures/ OR exp Self Report/ OR exp Patient Participation/ OR exp interview/ OR exp Focus Groups/ OR exp Qualitative Research/ OR exp Quality of Life/ OR exp Health Status/ OR Surveys and Questionnaires/ OR Attitude/ OR exp Health Status Indicators/ and (patient satisfaction/ or patient preference/ or Patient-Centered Care/) OR Outcome Assessment/ and (patient satisfaction/ or patient preference/ or Patient-Centered Care/ or Professional-Patient Relations/) OR Quality of Life/ and Health Status/ and patient satisfaction/ and (Outcome Assessment/ or Questionnaires/) OR exp Decision Making/ or Quality Indicators, Health Care/ or Feedback/ or Decision Making/ | Patient satisfaction OR (patient adj2 satisf*) OR patient reported experience measures OR PREM OR patient reported outcome measures OR PROM OR POEM OR HRPRO OR HRQL OR HRQoL OR QL OR QoL OR patient-reported outcome OR patient-reported experience measure* OR PRIM OR Patient-Reported Importance Measures OR ((Patient* or self or child or parent or proxy) adj2 (appraisal* or appraised or report* or rated or rating* or based or assessed or assessment* or experience*) OR public adj2 involvement*) OR Patient* adj2 ((report* adj2 outcome) or preference or Accept* or Maintain* or Sustain* or Impact or Engagement or perceiv* or interview*) OR perception of patient experience OR Surveys OR Questionnaires OR qualitative adj2 interview* OR focus groups OR health index* OR health indices OR health profile* OR subjective* OR self-rat*OR self adj2 rat* OR rat* scale OR (patient report* adj3 (outcome* or data)) OR (public report* adj3 hospital*) OR (star adj2 rating*) OR (subjective adj2 parameter*) OR( objective adj2 parameter*) OR perception* OR (subjective adj2 opinion*) OR (perceived adj2 (behavior OR behaviour)) OR (public* report* adj3 (outcome* OR data OR information OR care)).ti,ab. |
| --- | --- |

**Health Behavior Intervention**

| exp Diet/ OR exp Diet Therapy/ OR Nutrition Therapy/ OR exp Diet, Reducing/ OR exp Caloric Restriction/ OR exp Feeding Behavior/ OR exp Fasting/ OR exp Sleep/ OR exp Sleep Deprivation/ OR exp Sleep Wake Disorders/ OR exp Sleep Disorders, Circadian Rhythm/ OR Exercise/ OR exp Exercise Therapy/ OR Energy Intake/ OR Eating/ OR Hunger/ OR Satiation/ | (diet* OR nutrition* OR eat* OR food* OR eat* OR feed* OR hunger OR satiety OR satiation OR energy-intake OR energy intake OR Perceived eating behaviors OR Sleep* adj2 (Schedule* OR quality OR duration OR time or exten* OR time*) OR sleep disorder* OR sleep depriv* OR Subjective Sleep Parameters OR sleep quality OR self-reported sleep OR sleep OR Physical Activity OR workout OR train* OR run* OR swim* OR walk).ti,ab. |
| --- | --- |

**Timed Interventions**

| exp Time/ OR exp Time Factors/ OR exp Circadian Rhythm/ OR exp Chronobiology Disorders/ | (Time* OR schedul* OR morning OR afternoon OR evening OR intermittent OR Interval OR chrononutrition OR chrono adj2 nutrition).ti,ab. |
| --- | --- |

**EMBASE (OVID)**

**Patient Reported Outcomes**

| exp Patient Satisfaction/ OR exp patient reported outcome measures/ OR exp Self Report/ OR exp Patient Participation/ OR exp interview/ OR exp Focus Groups/ OR exp Qualitative Research/ OR exp Quality of Life/ OR exp Health Status/ OR Surveys and Questionnaires/ OR Attitude/ OR exp Health Status Indicators/ and (patient satisfaction/ or patient preference/ or Patient-Centered Care/) OR Outcome Assessment/ and (patient satisfaction/ or patient preference/ or Patient-Centered Care/ or Professional-Patient Relations/) OR Quality of Life/ and Health Status/ and patient satisfaction/ and (Outcome Assessment/ or Questionnaires/) OR exp Decision Making/ or Quality Indicators, Health Care/ or Feedback/ or Decision Making/ | Patient satisfaction OR (patient adj2 satisf*) OR patient reported experience measures OR PREM OR patient reported outcome measures OR PROM OR POEM OR HRPRO OR HRQL OR HRQoL OR QL OR QoL OR patient-reported outcome OR patient-reported experience measure* OR PRIM OR Patient-Reported Importance Measures OR ((Patient* or self or child or parent or proxy) adj2 (appraisal* or appraised or report* or rated or rating* or based or assessed or assessment* or experience*) OR public adj2 involvement*) OR Patient* adj2 ((report* adj2 outcome) or preference or Accept* or Maintain* or Sustain* or Impact or Engagement or perceiv* or interview*) OR perception of patient experience OR Surveys OR Questionnaires OR qualitative adj2 interview* OR focus groups OR health index* OR health indices OR health profile* OR subjective* OR self-rat*OR self adj2 rat* OR rat* scale OR (patient report* adj3 (outcome* or data)) OR (public report* adj3 hospital*) OR (star adj2 rating*) OR (subjective adj2 parameter*) OR( objective adj2 parameter*) OR perception* OR (subjective adj2 opinion*) OR (perceived adj2 (behavior OR behaviour)) OR (public* report* adj3 (outcome* OR data OR information OR care)).ti,ab. |
| --- | --- |

**Health Behavior Intervention**

| exp Diet/ OR exp Diet Therapy/ OR Nutrition Therapy/ OR exp Diet, Reducing/ OR exp Caloric Restriction/ OR exp Feeding Behavior/ OR exp Fasting/ OR exp Sleep/ OR exp Sleep Deprivation/ OR exp Sleep Wake Disorders/ OR exp Sleep Disorders, Circadian Rhythm/ OR Exercise/ OR exp Exercise Therapy/ OR Energy Intake/ OR Eating/ OR Hunger/ OR Satiation/ | (diet* OR nutrition* OR eat* OR food* OR eat* OR feed* OR hunger OR satiety OR satiation OR energy-intake OR energy intake OR Perceived eating behaviors OR Sleep* adj2 (Schedule* OR quality OR duration OR time or exten* OR time*) OR sleep disorder* OR sleep depriv* OR Subjective Sleep Parameters OR sleep quality OR self-reported sleep OR sleep OR Physical Activity OR workout OR train* OR run* OR swim* OR walk).ti,ab. |
| --- | --- |

**Timed Interventions**

| exp Time/ OR exp Time Factors/ OR exp Circadian Rhythm/ OR exp Chronobiology Disorders/ | (Time* OR schedul* OR morning OR afternoon OR evening OR intermittent OR Interval OR chrononutrition OR chrono adj2 nutrition).ti,ab. |
| --- | --- |

**Pubmed**

**Patient Reported Outcomes**

| Patient Satisfaction[mesh] OR patient reported outcome measures[mesh] OR Self Report[mesh] OR Patient Participation[mesh] OR interview[mesh] OR Focus Groups[mesh] OR Qualitative Research[mesh] OR Quality of Life[mesh] OR Health Status[mesh] OR Surveys and Questionnaires[mesh] OR Attitude[mesh] OR Health Status Indicators[mesh] and (patient satisfaction[mesh] or patient preference[mesh] or Patient-Centered Care[mesh]) OR Outcome Assessment[mesh] and (patient satisfaction[mesh] or patient preference[mesh] or Patient-Centered Care[mesh] or Professional-Patient Relations[mesh]) OR Quality of Life[mesh] OR Health Status[mesh] OR patient satisfaction[mesh] and (Outcome Assessment[mesh] or Questionnaires[mesh]) OR Decision Making[mesh] or Quality Indicators, Health Care[mesh] or Feedback[mesh] or Decision Making[mesh] | Patient satisfaction[tw] OR patient reported experience measures[tw] OR PREM[tw] OR patient reported outcome measures[tw] OR PROM[tw] OR POEM[tw] OR HRPRO[tw] OR HRQL[tw] OR HRQoL [tw] OR QL[tw] OR QoL[tw] OR patient-reported outcome[tw] OR patient-reported experience measure*[tw] OR PRIM[tw] OR Patient-Reported Importance Measures[tw] OR patient* appriasal[tw] OR patient* appraised[tw] OR patient* report*[tw] OR patient* rated[tw] OR patient* rating[tw] OR patient-based[tw] OR patient* based[tw] OR patient-assessed[tw] OR patient* assessed[tw] OR patient* experience[tw] OR self-appraisal[tw] OR self appraisal[tw] OR self-appraised[tw] OR self appraised OR self-report*[tw] OR self-rated[tw] OR self rated[tw] OR self-rating[tw] OR self rating[tw] OR self-based[tw] OR self based[tw] OR self-assessed[tw] OR self assessed[tw] OR self-assessment[tw] OR self assessment[tw] OR self-experience[tw] OR self experience[tw] OR child appraisal[tw] OR chil appraisal[tw] OR child appraised[tw] OR child report*[tw] OR child-rated[tw] OR child rated[tw] OR child rating[tw] OR child-rating[tw] OR child-based[tw] OR child based[tw] OR child assessed[tw] OR child-assessed[tw] OR child-assessment[tw] OR child assessment[tw] OR child experience[tw] OR parent appraisal[tw] OR parent appraised[tw] OR parent report*[tw] OR parent rated[tw] OR parent-rated[tw] OR parent-rating[tw] OR parent rating[tw] OR parent-based[tw] OR parent based[tw] OR parent assessed[tw] OR parent assessment[tw] OR parent-experience[tw] OR parent experience[tw] OR proxy appraisal[tw] OR proxy appraised[tw] OR proxy report*[tw] OR proxy rated[tw] OR proxy rating[tw] OR proxy based[tw] OR proxy assessed[tw] OR proxy assessment[tw] OR proxy experience[tw] OR public involvement*[tw] OR Patient* report* outcome*[tw] OR patient* preference[tw] OR patient Accept*[tw] OR Patient Maintain*[tw] or Patient Sustain*[tw] OR Patient Impact[tw] or Patient Engagement[tw] OR patient perceiv*[tw] OR patient interview*[tw] OR perception of patient experience[tw] OR Surveys[tw] OR Questionnaires[tw] OR qualitative interview*[tw] OR focus groups[tw] OR health index*[tw] OR health indices[tw] OR health profile*[tw] OR subjective*[tw] OR self-rat*[tw] OR rat* scale[tw] OR patient report* data[tw] OR public report*[tw] OR star rating*[tw] OR subjective parameter*[tw] OR objective parameter*[tw] OR perception*[tw] OR subjective opinion*[tw] OR perceived behavior[tw] OR perceived behaviour[tw] OR public* report* outcome*[tw] OR public* report* data[tw] |
| --- | --- |

**Health Behavior Intervention**

| Diet[mesh] OR Diet Therapy[mesh] OR Nutrition Therapy[mesh] OR Diet, Reducing[mesh] OR Caloric Restriction[mesh] OR Feeding Behavior[mesh] OR Fasting[mesh] OR Sleep[mesh] OR Sleep Deprivation[mesh] OR Sleep Wake Disorders[mesh] OR Sleep Disorders, Circadian Rhythm[mesh] OR Exercise[mesh] OR Exercise Therapy[mesh] OR Energy Intake[mesh] OR Eating[mesh] OR Hunger[mesh] OR Satiation[mesh] | diet*[tw] OR nutrition*[tw] OR eat*[tw] OR food*[tw] OR eat*[tw] OR feed*[tw] OR hunger[tw] OR satiety[tw] OR satiation[tw] OR energy-intake[tw] OR energy intake[tw] OR Perceived eating behaviors[tw] OR Sleep* schedule[tw] OR sleep* quality OR sleep* duration OR sleep* time OR sleep exten*[tw] OR sleep time*[tw] OR sleep disorder*[tw] OR sleep depriv*[tw] OR Subjective Sleep Parameters[tw] OR sleep quality[tw] OR self-reported sleep[tw] OR sleep[tw] OR Physical Activity[tw] OR workout[tw] OR train*[tw] OR run*[tw] OR swim*[tw] OR walk[tw] |
| --- | --- |

**Timed Interventions**

| Time[mesh] OR Time Factors[mesh] OR Circadian Rhythm[mesh] OR Chronobiology Disorders[mesh] | Time*[tw] OR schedul*[tw] OR morning[tw] OR afternoon[tw] OR evening[tw] OR intermittent[tw] OR Interval[tw] OR chrononutrition[tw] OR chrono nutrition[tw] |
| --- | --- |

**CINAHL (pls select in title and abstract next to the search strategy)**

**Patient Reported Outcomes**

| MH “Patient Satisfaction+” OR MH “patient reported outcome measures+” OR MH “Self Report+” OR MH “Patient Participation+” OR MH “interview+” OR MH “Focus Groups+” OR MH “Qualitative Research+” OR MH “Quality of Life+” OR MH “Health Status+” OR MH “Surveys and Questionnaires+” OR MH “Attitude” OR MH “Health Status Indicators+” and MH “patient satisfaction” OR MH “patient preference” OR MH “Patient-Centered Care” OR MH “Outcome Assessment” OR MH “patient satisfaction” OR MH “patient preference” OR MH “Patient-Centered Care” OR MH “Professional-Patient Relations” OR MH “Quality of Life” OR MH “Health Status” OR MH “patient satisfaction” OR MH “Outcome Assessment” OR MH “Questionnaires” OR MH “Decision Making+” OR MH “Quality Indicators, Health Care” OR MH “Feedback” OR MH “Decision Making” | Patient satisfaction OR (patient N2 satisf*) OR patient reported experience measures OR PREM OR patient reported outcome measures OR PROM OR POEM OR HRPRO OR HRQL OR HRQoL OR QL OR QoL OR patient-reported outcome OR patient-reported experience measure* OR PRIM OR Patient-Reported Importance Measures OR ((Patient* or self or child or parent or proxy) N2 (appraisal* or appraised or report* or rated or rating* or based or assessed or assessment* or experience*) OR public N2 involvement*) OR Patient* N2 ((report* N2 outcome) or preference or Accept* or Maintain* or Sustain* or Impact or Engagement or perceiv* or interview*) OR perception of patient experience OR Surveys OR Questionnaires OR qualitative N2 interview* OR focus groups OR health index* OR health indices OR health profile* OR subjective* OR self-rat*OR self N2 rat* OR rat* scale OR (patient report* N3 (outcome* or data)) OR (public report* N3 hospital*) OR (star N2 rating*) OR (subjective N2 parameter*) OR (objective N2 parameter*) OR perception* OR (subjective N2 opinion*) OR (perceived N2 (behavior OR behaviour)) OR (public* report* N3 (outcome* OR data OR information OR care)) |
| --- | --- |

**Health Behavior Intervention**

| MH “Diet+” OR MH “Diet Therapy+” OR MH “Nutrition Therapy+” OR MH “Diet, Reducing+” OR MH “Caloric Restriction+” OR MH “Feeding Behavior+” OR MH “Fasting+” OR MH “Sleep+” OR MH “Sleep Deprivation+” OR MH “Sleep Wake Disorders+” OR MH “Sleep Disorders, Circadian Rhythm+” OR MH “Exercise” OR MH “Exercise Therapy” OR MH “Energy Intake” OR MH “Eating” OR MH “Hunger” OR MH “Satiation” | TI (diet* OR nutrition* OR eat* OR food* OR eat* OR feed* OR hunger OR satiety OR satiation OR energy-intake OR energy intake OR Perceived eating behaviors OR Sleep* adj2 (Schedule* OR quality OR duration OR time or exten* OR time*) OR sleep disorder* OR sleep depriv* OR Subjective Sleep Parameters OR sleep quality OR self-reported sleep OR sleep OR Physical Activity OR workout OR train* OR run* OR swim* OR walk) |
| --- | --- |

**Timed Interventions**

| MH “Time+” OR MH “Time Factors+” OR MH “Circadian Rhythm+” OR MH “Chronobiology Disorders+” | TI (Time* OR schedul* OR morning OR afternoon OR evening OR intermittent OR Interval OR chrononutrition OR chrono N2 nutrition) |
| --- | --- |

**Psychinfo**

**Patient Reported Outcomes**

| DE “Patient Satisfaction” OR DE “patient reported outcome measures” OR DE “Self Report” OR DE “Patient Participation” OR DE “interview” OR DE “Focus Groups” OR DE “Qualitative Research” OR DE “Quality of Life” OR DE “Health Status” OR DE “Surveys and Questionnaires” OR DE “Attitude” OR DE “Health Status Indicators” and DE “patient satisfaction” OR DE “patient preference” OR DE “Patient-Centered Care” OR DE “Outcome Assessment” OR DE “patient satisfaction” OR DE “patient preference” OR DE “Patient-Centered Care” OR DE “Professional-Patient Relations” OR DE “Quality of Life” OR DE “Health Status” OR DE “patient satisfaction” OR DE “Outcome Assessment” OR DE “Questionnaires” OR DE “Decision Making” OR DE “Quality Indicators, Health Care” OR DE “Feedback” OR DE “Decision Making” | TI (Patient satisfaction OR (patient N2 satisf*) OR patient reported experience measures OR PREM OR patient reported outcome measures OR PROM OR POEM OR HRPRO OR HRQL OR HRQoL OR QL OR QoL OR patient-reported outcome OR patient-reported experience measure* OR PRIM OR Patient-Reported Importance Measures OR ((Patient* or self or child or parent or proxy) N2 (appraisal* or appraised or report* or rated or rating* or based or assessed or assessment* or experience*) OR public N2 involvement*) OR Patient* N2 ((report* N2 outcome) or preference or Accept* or Maintain* or Sustain* or Impact or Engagement or perceiv* or interview*) OR perception of patient experience OR Surveys OR Questionnaires OR qualitative N2 interview* OR focus groups OR health index* OR health indices OR health profile* OR subjective* OR self-rat*OR self N2 rat* OR rat* scale OR (patient report* N3 (outcome* or data)) OR (public report* N3 hospital*) OR (star N2 rating*) OR (subjective N2 parameter*) OR (objective N2 parameter*) OR perception* OR (subjective N2 opinion*) OR (perceived N2 (behavior OR behaviour)) OR (public* report* N3 (outcome* OR data OR information OR care))) |
| --- | --- |

**Health Behavior Intervention**

| DE “Diet” OR DE “Diet Therapy” OR DE “Nutrition Therapy” OR DE “Diet, Reducing” OR DE “Caloric Restriction” OR DE “Feeding Behavior” OR DE “Fasting” OR DE “Sleep” OR DE “Sleep Deprivation” OR DE “Sleep Wake Disorders” OR DE “Sleep Disorders, Circadian Rhythm” OR DE “Exercise” OR DE “Exercise Therapy” OR DE “Energy Intake” OR DE “Eating” OR DE “Hunger” OR DE “Satiation” | TI (diet* OR nutrition* OR eat* OR food* OR eat* OR feed* OR hunger OR satiety OR satiation OR energy-intake OR energy intake OR Perceived eating behaviors OR Sleep* adj2 (Schedule* OR quality OR duration OR time or exten* OR time*) OR sleep disorder* OR sleep depriv* OR Subjective Sleep Parameters OR sleep quality OR self-reported sleep OR sleep OR Physical Activity OR workout OR train* OR run* OR swim* OR walk) |
| --- | --- |

**Timed Interventions**

| DE “Time” OR DE “Time Factors” OR DE “Circadian Rhythm” OR DE “Chronobiology Disorders+” | TI (Time* OR schedul* OR morning OR afternoon OR evening OR intermittent OR Interval OR chrononutrition OR chrono N2 nutrition) |
| --- | --- |

**SCOPUS (no mesh words apply)**

**Patient Reported Outcomes**

| “Patient satisfaction” OR (patient pre/2 satisf*) OR “patient reported experience measures” OR PREM OR “patient reported outcome measures” OR PROM OR POEM OR HRPRO OR HRQL OR HRQoL OR QL OR QoL OR “patient-reported outcome” OR “patient-reported experience measure*” OR PRIM OR “Patient-Reported Importance Measures” OR Patient* pre/2 appraisal or self pre/2 appraisal or child pre/2 appraisal or parent pre/2 appraisal or proxy pre/2 appraisal or patient pre/2 appraised or self re/2 appraised or child pre/2 appraised or parent pre/2 appraised or proxy pre/2 appraised or patient pre/2 report* or self pre/2 report or child pre/2 report or parent pre/2 report or proxy pre/2 report or patient pre/2 rated or self pre/2 rated or child pre/2 rated or parent pre/2 rated or proxy pre/2 rated or patient pre/2 rating* or self pre/2 rating or child pre/2 rating or parent pre/2 rating or proxy pre/2 rating or patient pre/2 based or self pre/2 based or child pre/2 based or parent pre/2 based or proxy pre/2 based or patient pre/2 assessed or self pre/2 assessed or child pre/2 assessed or parent pre/2 assessed or proxy pre/2 assessed or patient pre/2 assessment* or self pre/2 assessment or child pre/2 assessment or parent pre/2 assessment or proxy pre/2 assessment or patient pre/2 experience* or self pre/2 experience or child pre/2 experience or parent pre/2 experience or proxy pre/2 experience OR public pre/2 involvement* OR Patient* pre/2 “reported outcome” or patient* pre/2 preference or patient* pre/2 Accept* or patient* pre/2 Maintain* or patient* pre/2 Sustain* or patient* pre/2 Impact or patient* pre/2 Engagement or patient* pre/2 perceiv* or patient* pre/2 interview* OR “perception of patient experience” OR Surveys OR Questionnaires OR qualitative pre/2 interview* OR “focus groups” OR “health index*” OR “health indices” OR “health profile*” OR subjective* OR self-rat*OR self pre/2 rat* OR rat* scale OR “patient report* outcome” or “patient reported data” OR public report* pre/2 hospital* OR star pre/2 rating* OR subjective pre/2 parameter* OR objective pre/2 parameter* OR perception* OR subjective pre/2 opinion* OR perceived pre/2 behavior OR perceived pre/2 behaviour OR “public* report* pre/3 outcome*” OR “public* report* pre/3 data” OR information OR care |
| --- |

**Health Behavior Intervention**

| diet* OR nutrition* OR eat* OR food* OR eat* OR feed* OR hunger OR satiety OR satiation OR energy-intake OR “energy intake” OR “Perceived eating behaviors” OR Sleep* pre/2 Schedule* OR sleep* pre/2 quality OR sleep* pre/2 duration OR sleep* pre/2 time or sleep* pre/2 exten* OR sleep* pre/2 time* OR “sleep disorder*” OR “sleep depriv*” OR “Subjective Sleep Parameters” OR “sleep quality” OR “self-reported sleep” OR sleep OR “Physical Activity” OR workout OR train* OR run* OR swim* OR walk |
| --- |

**Timed Interventions**

| Time* OR schedul* OR morning OR afternoon OR evening OR intermittent OR Interval OR chrononutrition OR chrono N2 nutrition |
| --- |

**Web of Science (no mesh words) AND GOOGLE SCHOLAR**

**Patient Reported Outcomes**

| Patient satisfaction OR patient reported experience measures OR PREM OR patient reported outcome measures OR PROM OR POEM OR HRPRO OR HRQL OR HRQoL OR QL OR QoL OR patient-reported outcome OR patient-reported experience measure OR PRIM OR Patient-Reported Importance Measures OR Patient appraisal or self-appraisal or child appraisal or parent appraisal or proxy appraisal or patient appraised or self-appraised or child appraised or parent appraised or proxy appraised or patient report* or self-report or child report or parent report or proxy report or patient rated or self-rated or child rated or parent rated or proxy rated or patient rating* or self-rating or child rating or parent rating or proxy rating or patient based or self-based or child-based or parent-based or proxy-based or patient assessed or self-assessed or child assessed or parent assessed or proxy assessed or patient assessment* or self-assessment or child assessment or parent assessment or proxy assessment or patient experience* or self-experience or child experience or parent experience or proxy experience OR public involvement* OR Patient* reported outcome or patient* preference or patient* Accept* or patient* Maintain* or patient* Sustain* or patient* Impact or patient* Engagement or patient* perceiv* or patient* interview* OR perception of patient experience OR Surveys OR Questionnaires OR qualitative interview* OR focus groups OR health index* OR health indices OR health profile* OR subjective* OR self-rat* OR rat* scale OR patient report* outcome or patient reported data OR public report* hospital* OR star rating* OR subjective parameter* OR objective parameter* OR perception* OR subjective opinion* OR perceived behavior OR perceived behaviour OR public* report* outcome* OR public* report* data OR information OR care |
| --- |

**Health Behavior Intervention**

| diet* OR nutrition* OR eat* OR food* OR eat* OR feed* OR hunger OR satiety OR satiation OR energy-intake OR energy intake OR Perceived eating behaviors OR Sleep* Schedule* OR sleep* quality OR sleep* duration OR sleep* time or sleep* exten* OR sleep* time* OR sleep disorder* OR sleep depriv* OR Subjective Sleep Parameters OR sleep quality OR self-reported sleep OR sleep OR Physical Activity OR workout OR train* OR run* OR swim* OR walk |
| --- |

**Timed Interventions**

| Time* OR schedul* OR morning OR afternoon OR evening OR intermittent OR Interval OR chrononutrition OR chrono-nutrition |
| --- |
