# Supplementary material for: Effect of timed exercise interventions on patient-reported outcome measures: A systematic review
Source: PLoS One. 2025 May 7;20(5):e0321526. doi: 10.1371/journal.pone.0321526 (PMC12057914; doi:10.1371/journal.pone.0321526)
Supplement: S3 Appendix — (DOCX) [file pone.0321526.s003.docx]

**Appendix C: List of studies reviewed at full-text stage and reasons for exclusion/inclusion**

As described in the manuscript, this review originated as a broader review inclusive of timed physical activity, timed eating/dietary patterns, and sleep timing/patterns. Therefore the original search included studies related to each of these domains.

Our search yielded 30 articles related to timed exercise which were reviewed as full-texts for inclusion in the present review. After full-text review of these 30 articles, 14 were excluded for the following reasons:

|  | **Study URL** | **Reasons for exclusion** |
| --- | --- | --- |
| 1 | doi.10.1152/japplphysiol.00765.2018 | Not Timed study |
| 2 | doi.10.1046/j.1440-1819.2001.00816.x | Not Timed study |
| 3 | doi.10.1249/MSS.0000000000000755 | Not Timed study |
| 4 | doi.org/10.1123/ijsnem.2019-0237 | Not Timed study |
| 5 | doi. 10.1007/s00421-011-2034-9 | Not Timed study |
| 6 | doi.org/10.1007/s00421-019-04280-w | Not Timed study |
| 7 | doi. 10.1111/j.1365-2869.2010.00874.x | Not Timed study |
| 8 | doi.org/10.1123/ijspp.2016-0626 | Not Timed study |
| 9 | [doi.org/10.1539/joh.40.37](https://doi.org/10.1539/joh.40.37) | Case study (Only one participant) |
| 10 | doi.org/10.1016/j.physbeh.2022.113906 | Not Timed study |
| 11 | doi: 10.1093/sleep/23.1.1f | Not Timed study |
| 12 | doi.org/10.1080/02640410801930150 | No PROM |
| 13 | doi.10.1152/japplphysiol.01365.2011. | No PROM |
| 14 | doi: 10.1519/1533-4287(2001)015<0127:TEOAMA>2.0.CO;2 | Not on adults |

After full-text review of these 30 articles, 16 were included in the review for the following reasons:

|  | **Study URL** | **Reasons for inclusion** |
| --- | --- | --- |
| 1 | doi:10.1007/s41782-021-00187-9 | Timed Exercise, with 2 different time of day tested on PROMs of adults |
| 2 | doi: 10.1556/2060.104.2017.1.6 | Timed Exercise, with 2 different time of day tested on PROMs of adults |
| 3 | doi: 10.1016/j.appet.2019.104411 | Timed Exercise, with 2 different time of day tested on PROMs of adults |
| 4 | doi: 10.1016/j.appet.2022.106422 | Timed Exercise, with 2 different time of day tested on PROMs of adults |
| 5 | <https://pubmed.ncbi.nlm.nih.gov/28011947/> | Timed Exercise, with 2 different time of day tested on PROMs of adults |
| 6 | doi: 10.1093/sleep/27.8.1542 | Timed Exercise, with 2 different time of day tested on PROMs of adults |
| 7 | doi: 10.1016/j.sleep.2017.03.014 | Timed Exercise, with 2 different time of day tested on PROMs of adults |
| 8 | doi: 10.1123/ijsnem.14.5.501 | Timed Exercise, with 2 different time of day tested on PROMs of adults |
| 9 | doi: 10.1002/nur.4770080310. | Timed Exercise, with 2 different time of day tested on PROMs of adults |
| 10 | doi: 10.1080/02640414.2013.873139 | Timed Exercise, with 2 different time of day tested on PROMs of adults |
| 11 | doi: 10.1519/JSC.0b013e3181a05564 | Timed Exercise, with 2 different time of day tested on PROMs of adults |
| 12 | doi: 10.1016/j.appet.2021 | Timed Exercise, with 2 different time of day tested on PROMs of adults |
| 13 | doi: 10.1080/07420528.2019.1592184 | Timed Exercise, with 2 different time of day tested on PROMs of adults |
| 14 | doi: 10.1177/0891988720924709. | Timed Exercise, with 2 different time of day tested on PROMs of adults |
| 15 | doi: 10.1080/07420528.2021.1935988. | Timed Exercise, with 2 different time of day tested on PROMs of adults |
| 16 | doi: 10.1016/j.sleep.2011.02.007 | Timed Exercise, with 2 different time of day tested on PROMs of adults |
